# Zinc protects against diabetes-induced pathogenic changes in the aorta: roles of metallothionein and nuclear factor (erythroid-derived 2)-like 2

**DOI:** 10.1186/1475-2840-12-54

**Published:** 2013-03-28

**Authors:** Xiao Miao, Yonggang Wang, Jian Sun, Weixia Sun, Yi Tan, Lu Cai, Yang Zheng, Guanfang Su, Quan Liu, Yuehui Wang

**Affiliations:** 1 Department of Ophthalmology, the Second Hospital of Jilin University, 218 Ziqiang Street, Changchun, 130041, China; 2 The First Hospital of Jilin University, 71 Xinmin Street, Changchun, 130021, China; 3 Kosair Children Hospital Research Institute at the Department of Pediatrics, University of Louisville, Louisville, 40202, USA; 4 Chinese-American Research Institute for Diabetic Complications, Wenzhou Medical College, Wenzhou, 325035, China; 5 Departments of Radiation Oncology and Pharmacology and Toxicology, University of Louisville, Louisville, 40202, USA

**Keywords:** Zinc, Vascular damage, Diabetes, Oxidative stress, Nrf2, Metallothionein

## Abstract

**Background:**

Cardiovascular diseases remain a leading cause of the mortality world-wide, which is related to several risks, including the life style change and the increased diabetes prevalence. The present study was to explore the preventive effect of zinc on the pathogenic changes in the aorta.

**Methods:**

A genetic type 1 diabetic OVE26 mouse model was used with/without zinc supplementation for 3 months. To determine gender difference either for pathogenic changes in the aorta of diabetic mice or for zinc protective effects on diabetes-induced pathogenic changes, both males and females were investigated in parallel by histopathological and immunohistochemical examinations, in combination of real-time PCR assay.

**Results:**

Diabetes induced significant increases in aortic oxidative damage, inflammation, and remodeling (increased fibrosis and wall thickness) without significant difference between genders. Zinc treatment of these diabetic mice for three months completely prevented the above pathogenic changes in the aorta, and also significantly up-regulated the expression and function of nuclear factor (erythroid-derived 2)-like 2 (Nrf2), a pivotal regulator of anti-oxidative mechanism, and the expression of metallothionein (MT), a potent antioxidant. There was gender difference for the protective effect of zinc against diabetes-induced pathogenic changes and the up-regulated levels of Nrf2 and MT in the aorta.

**Conclusions:**

These results suggest that zinc supplementation provides a significant protection against diabetes-induced pathogenic changes in the aorta without gender difference in the type 1 diabetic mouse model. The aortic protection by zinc against diabetes-induced pathogenic changes is associated with the up-regulation of both MT and Nrf2 expression.

## Introduction

Cardiovascular disease is a leading cause of mortality. The causes of cardiovascular diseases are multifaceted, including environmental pollution [1,2] and life style changes such as the lack of physical activity and the increased intake of Western foods that include over-nutrition and trace elemental dyshomeostasis [3,4].

Cardiovascular diseases are sex difference. For instance, men have an increased incidence and severity of most cardiovascular diseases, including atherosclerosis, myocardial infarction, dilated cardiomyopathy, and heart failure, with the exception of hypertension that is higher in women [1-4]. However, the preventive effects that make females with a low incidence of cardiovascular diseases compared to males were diminished under diabetic conditions [5]. This may be because the vascular gender peculiarities. For instance, animal studies showed that endothelium-intact thoracic aortic rings from age-matched male and female Sprague–Dawley rats were responsive to insulin, by showing the relaxation. The hyperglycemia was found able to inhibit the response of aortic rings to insulin and apparently the female vascular endothelium is more sensitive to the toxic effect of hyperglycemia than the male vascular endothelium [6]. Human studies also supports the concept that women who progressed from normoglycemia to pre-diabetes or hyperglycemia have a greater endothelial dysfunction, more hypertension, and a greater degree of fibrinolysis/thrombosis than men [7]. However, mechanisms by which diabetes impacts more female than male remain unclear [6,7].

Zinc (Zn) is one of the important essential trace metals that are required for many cell events. Zn is not only an important nutrient, cofactor of numerous enzymes and transcription factors, but also acts as intracellular signaling mediator [8]. So far, more than 300 catalytically active Zn metalloproteinase and more than 2000 Zn dependent transcription factors have been recognized. Therefore, Zn dyshomeostasis such as Zn deficiency is associated with various chronic pathogeneses, including vascular diseases [9]. For instance, Zn deficiency in endothelial cells potentiates the inflammatory response mediated by certain lipids and cytokines, possibly via mechanisms associated with increased cellular oxidative stress [10,11]; however, Zn supplementation protects vascular system from oxidative damage [12,13].

One of the mechanisms by which Zn supplementation protects vascular system from oxidative damage may include the up-regulation of NF-E2-related factor 2 (Nrf2) expression and function [14,15]. The antioxidant responsive element (ARE) is a cis-acting regulatory element of genes encoding phase II detoxification enzymes and antioxidant proteins, such as NAD(P)H: quinone oxidoreductase 1 (NQO-1), HO-1, glutathione S-transferases, and glutamate-cysteine ligase. Nrf2 regulates a wide array of ARE-driven genes in various cell types. The DNA binding sequence of Nrf2 and ARE sequence are very similar so that Nrf2 binds to the ARE sites leading to up-regulation of downstream genes. Therefore, the Nrf2-ARE pathway is important in the cellular antioxidant defense system to protect the cell and tissue from oxidative stress, including diabetes [16,17].

Another important potential mechanism may be the induction of metallothionein (MT) expression [18,19]. MTs are cysteine-rich metal-binding proteins with several biological roles including antioxidant property. We and others have indicated the significant protection of MT against diabetes and diabetes-induced cardiovascular damage [19-22]. MT is ubiquitously expressed in mammalian tissues and also highly inducible by a variety of reagents such as Zn; therefore, the protective effect of Zn supplementation on diabetic heart and kidney was noticed before [18,23,24].

Therefore, the present study was first to explore whether diabetes-induced pathogenic changes in the aorta can be prevented by Zn supplementation, and to compare whether there is a difference between female and male for the preventive effect of Zn supplementation on diabetes-induced pathogenic changes in the aorta. Then the possible mechanisms by which Zn prevents the aorta from diabetes-induced pathogenic changes were explored by analyzing Nrf2’s expression and function, and also MT expression.

## Materials and methods

### Animals

OVE26 type 1 diabetic mice with FVB background were have been used in our previous studies [25,26]. Mice were housed in the University of Louisville Research Resources Center at 22°C with a 12-h light/dark cycle and provided with free access to standard rodent chow and tap water. All animal procedures were approved by the Institutional Animal Care and Use Committee, which is certified by the American Association for Accreditation of Laboratory Animal Care.

These OVE26 mice normally develop severe hyperglycemia 2 – 3 weeks after birth, and develop macro proteinuria significantly at 3 month of age [27,28]. Three months old male and female OVE26 mice were randomly divided into two groups: diabetes (DM) and diabetes supplement with Zn (DM/Zn) with 6 mice per group of each gender. Age- and sex-matched FVB mice were also randomly divided into two groups: non-diabetic control (control) and Zn control (Zn) with 6 mice in each group of each gender. For Zn and DM/Zn mice, Zn supplementation was given by gavage at 5 mg ZnSO_4_/kg every other day for 3 months while control and DM group mice were administered with equal amounts of saline. Volume of ZnSO_4_ solution was calculated based on individual rat body weight (0.1 ml ZnSO_4_/g body weight). All mice from both control and DM groups of both male and female were sacrificed at 6 months of age (i.e. Zn supplementation for 3 months).

### Aorta preparation and histopathological examination

After anesthesia, thorax was opened and descending thoracic aortas were isolated carefully and cleaned of surrounding fat and connective tissue. Aortas tissues were fixed in 10% buffered formalin and then cut into ring segments (2 – 3 mm in length) for being dehydrated in graded alcohol series, cleared with xylene, embedded in paraffin, and sectioned at 5 μm thickness for pathological and immunohistochemical staining. Histological evaluation of aorta was performed by H&E staining with Image Pro Plus 6.0 software for measuring the means of the tunica media width size as the thickness of aortic tunica media.

Paraffin sections (5 μm thickness) from aortic tissues were dewaxed and incubated with 1X Target Retrieval Solution (Dako, Carpinteria, CA) in a microwave oven for 15 min at 98°C for antigen retrieval, with 3% hydrogen peroxide for 15 min at room temperature, and then with 5% animal serum for 30 min, respectively. These sections were incubated with primary antibodies against connective tissue growth factor (CTGF) and transforming growth factor (TGF)-β1 at 1:100 dilution (Santa Cruz Biotechnology, Santa Cruz, CA), 3-nitrotyrosine (3-NT) at 1:400 dilution (Millipore, Billerica, CA), 4-hydroxy-2-nonenal (4-HNE) at 1:400 dilution (Alpha Diagnostic International, San Antonio, TX), Collagen IV at 1:200 dilution (Santa Cruz Biotechnology, Santa Cruz, CA), plasminogen activator inhibitor-1 (PAI-1) at 1:100 dilution (BD Bioscience, San Jose, CA), tumor necrosis factor-α (TNF-α, Abcam, Cambridge, MA) at 1:100 dilution, nuclcar- associated antigen ki-67 at 1:400 dilution (Abcam, Cambridge, MA), MT at 1:100 dilution (DakoInc, Carpinteria, CA), NADPH quinine oxidoreductase (NQO-1) at 1:200 dilution (Cell Signaling, MA), Nrf2at 1:100 dilution, and vascular cell adhesion molecule 1 (VCAM-1) at 1:100 dilution (both from Santa Cruz Biotechnology) overnight at 4°C. After sections were washed with PBS, they were incubated with horseradish peroxidase conjugated secondary antibodies (1:300 – 400 dilutions with PBS) or Cy3-coupled goat anti-rabbit IgG secondary antibody for 2 h in room temperature. For the color development of immunohistochemical staining sections were treated with peroxidase substrate DAB kit (Vector Laboratories, Inc. Burlingame, CA) and counterstained with hematoxylin. For immunofluorescent staining sections were stained with DAPI at 1:1000 dilution to localize the nucleus.

For quantitative analysis of these immunohistochemical staining, three sections at interval of 10 sections from each aorta (per mouse) were selected and at least five high-power fields were randomly captured per section. Image Pro Plus 6.0 software was used to transfer the interesting area staining density to an integrated optical density (IOD) that was divided by the area size of interest to reflect the staining level of the area of interest, and the ratio of IOD/area size in experimental group was presented as a fold relative to that of control. For Ki-67 staining, the positively stained nuclei were counted randomly in five microscopic fields at least of each of the three slides per aorta (mouse) under light microscopy. The percentage of positive staining nuclei in total 100 nuclei was presented.

### Sirius-red staining for collagen

Aortic fibrosis was reflected by Sirius-red staining for collagen, as described in our previous study [21]. Briefly, 5 μm tissue sections were used for Sirius-red staining with 0.1% Sirius-red F3BA and 0.25% Fast Green FCF. Sections stained for Sirius-red then were assessed for the proportion of collagen using a Nikon Eclipse E600 microscopy system.

### TUNEL staining

Terminal deoxynucleotidyl-transferase-mediated dUTP-nick-end labeling (TUNEL) staining was performed on formalin-fixed, paraffin-embedded sections with Peroxidase In Situ Apoptosis Detection Kit S7100 (Millipore, Billerica, MA) according to the instructions. The positively stained apoptotic cells were counted randomly in five microscopic fields at least of each of the three slides per aorta (mouse) under light microscopy. The percentage of TUNEL positive cells relative to 100 nuclei was presented.

### Real-time qPCR

Collected aortas were snap frozen in liquid nitrogen and kept at - 80°C. Total RNA was extracted using the TRIzol Reagent (Invitrogen, USA). RNA concentrations and purities were quantified using a Nanodrop ND-1000 spectrophotometer. First-strand complimentary DNA (cDNA) was synthesized from total RNA according to manufacturer’s protocol from the RNA PCR kit (Promega, Madison, WI). Reverse transcription was performed using 0.5 μg of total RNA in 12.5 μl of the solution containing 4 μl 25 mM MgCl_2_, 4 μl AMV reverse transcriptase 5 X buffer, 2 μl dNTP, 0.5 μl RNase inhibitor, 1 μl of AMV reverse transcriptase, and 1 μl of oligodT primer, which were added with nuclease-free water to make a final volume of 20 μl. Reaction system was run at 42°C for 50 min and 95°C for 5 min. Primers of TNF-α, Nrf2, MT, NQO-1, and β-actin were purchased from Applied Biosystems (Carlsbad, CA). Real-time quantitative PCR (qPCR) was carried out in a 20 μl reaction buffer that included 10 μl of TaqMan Universal PCR Master Mix, 1 μl of primer, 9 μl of cDNA with the ABI 7300 Real-Time PCR system. The fluorescence intensity of each sample was measured at each temperature change to monitor amplification of the target gene. The comparative cycle time (CT) was used to determine fold differences between samples.

### Statistical analysis

Data were collected from several animals (n = 6) and presented as means ± SD. We used Image Pro Plus 6.0 software to measure the pathological changes, as described above. Comparisons were performed by one-way ANOVA for the different groups, followed by post hoc pairwise repetitive comparisons using Tukey’s test with Origin 7.5 Lab data analysis and graphing software. Statistical significance was considered as P <0.05.

## Results

### Preventive effect of Zn on diabetes-induced aortic fibrosis

**Figure 1:**
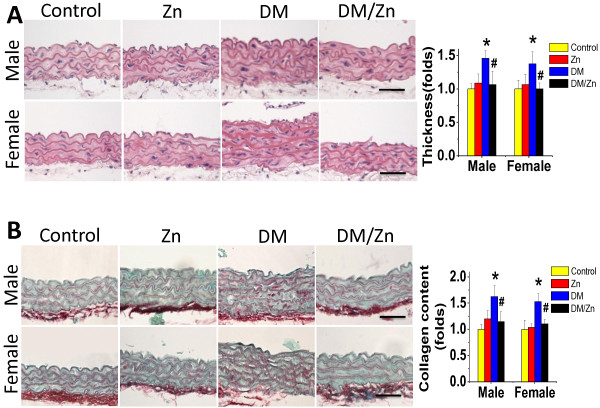
**Preventive effect of Zn on diabetes-induced aortic pathological changes.** The pathogenic changes of aortas were examined by H&E staining (**A**), and Sirius-red staining for collagen accumulation (**B**), followed with semi-quantitative analysis. Data were presented as means ± SD (n = 6). *, p < 0.05 vs. Corresponding Control; #, p < 0.05 vs. Corresponding DM. Bar = 50 μM.

OVE26 transgenic diabetic mice and age-matched FVB mice both male and female were treated with Zn for 3 months. Aortas were examined pathologically with H&E staining (Figure 1A), which showed the increase in tunic media thickness significantly in diabetes group both male and female. Similarly Sirius-red staining revealed an increased collagen accumulation in aortic tunica media of diabetic mice (Figure 1B). Both pathological alterations were completely prevented by Zn supplementation in DM/Zn group without gender difference.

**Figure 2:**
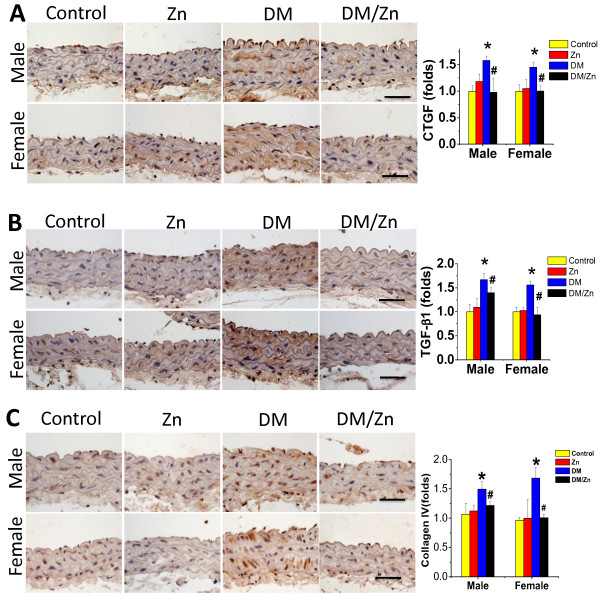
**Preventive effect of Zn on diabetes-induced aortic fibrosis.** Aortic fibrosis was examined by immunohistochemical staining for the expression of CTGF (**A**), TGF-β1 (**B**) and Collagen IV (**C**), followed with semi-quantitative analysis. Data were presented as means ± SD (n = 6). *, p < 0.05 vs. Corresponding Control; #, p < 0.05 vs. Corresponding DM. Bar = 50 μM.

To further examine the preventive effect of Zn on diabetes-induced aortic fibrosis, immunohistochemical staining showed the increased expression of pro-fibrotic mediators, CTGF and TGF-β1, and collagen IV accumulation, in aortic tunica media of diabetic mice without gender difference (Figure 2). Supplementation with Zn could completely prevent these fibrotic responses in the aortas of diabetic mice (DM/Zn group).

### Preventive effect of Zn on diabetes-induced aortic inflammation and oxidative damage

**Figure 3:**
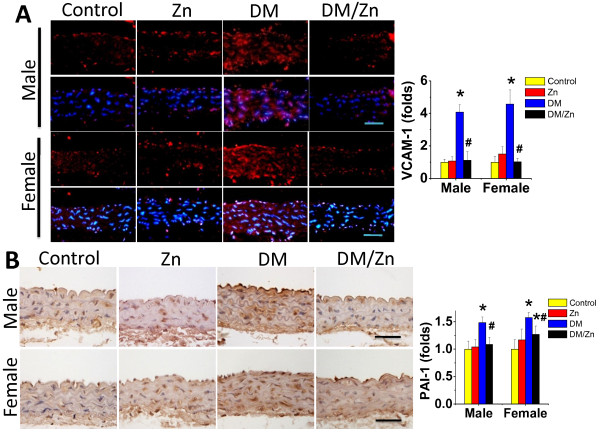
**Preventive effect of Zn on diabetes-induced aortic inflammation.** Aortic inflammation was examined by immunofluorescent staining for the expression of VCAM-1(**A**) (red) and immunohistochemical staining for the expression of PAI-1 (**B**), followed by semi-quantitative analysis. Data were presented as means ± SD (n = 6).*, p < 0.05 vs. Corresponding Control; #, p < 0.05 vs. Corresponding DM. Bar = 50 μM.

**Figure 4:**
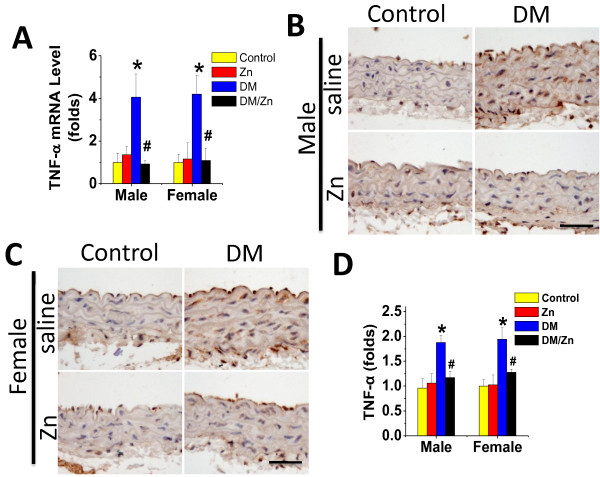
**Preventive effect of Zn on diabetes-induced aortic TNF-α expression.** Aortic expression of TNF-α was examined by real-time PCR for its mRNA level (**A**) and immunohistochemical staining for its protein expression in aortic tunica media (**B** for male and **C** for female), followed by semi-quantitative analysis (**D** for both male and female). Data were presented as means ± SD (n = 6).*, p < 0.05 vs. Corresponding Control; #, p < 0.05 vs. Corresponding DM. Bar = 50 μM.

Immunohistochemical staining showed a significant increase in aortic expression of inflammatory markers VCAM-1 (Figure 3A) and PAI-1 (Figure 3B) in the aortic tunica media of diabetic mice for both genders. Supplementation with Zn can completely prevent the inflammation response in aortas of diabetic mice (DM/Zn group). We also examined the expression of TNF-α as an important inflammatory factor at mRNA (Figure 4A) and protein (Figure 4B-D) levels, which shows that a significant increase in aortic expression of TNF-α at both mRNA and protein levels in the aortic tunica media of diabetic mice without gender difference. This effect was completely prevented by supplementation with Zn in diabetic mice (DM/Zn group).

**Figure 5:**
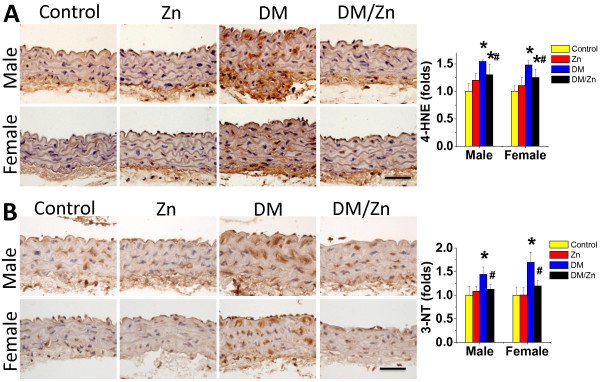
**Preventive effect of Zn on diabetes-induced aortic oxidative damage.** The oxidative damage was examined by immunohistochemical staining for the accumulation of 4-HNE (**A**) and 3-NT (**B**), followed with semi-quantitative analysis. Data were presented as means ± SD (n = 6).*, p < 0.05 vs. Corresponding Control; #, p < 0.05 vs. Corresponding DM. Bar = 50 μM.

In addition, a significantly increased accumulation of oxidative and nitrative damage, 4-HNE (Figure 5A) and 3-NT (Figure 5B), was also evident in aortic tunica media of diabetic mice, but not in Zn-treated diabetic mice (DM/Zn group).

### Preventive effect of Zn on diabetes-induced aortic apoptotic cell death and proliferation

**Figure 6:**
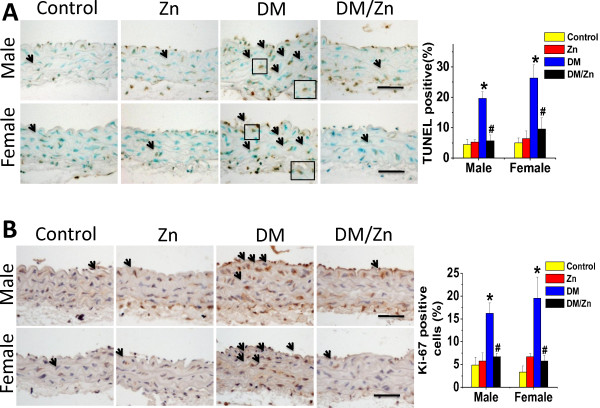
**Diabetes induced aortic apoptosis and proliferation increased.** The apoptotic cell was examined by TUNEL staining (**A**), followed with semi-quantitative analysis. And the proliferation of aortic tunica media was examined by immunohistochemical staining for ki-67 positive cells (**B**), followed with semi-quantitative analysis. Data were presented as means ± SD (n = 6).*, p < 0.05 vs. Corresponding Control; #, p < 0.05 vs. Corresponding DM. Bar = 50 μM.

Diabetes was found to induce an increase in the apoptotic cell death, reflected by TUNEL positive cells (Figure 6A) and cell proliferation, reflected by Ki-67 positive nuclear (Figure 6B) in the aorta of both male and female diabetic mice (DM group), but not in Zn-treated diabetic mice (DM/Zn group).

### Up-regulation of Nrf2 expression and its downstream antioxidant gene expression by Zn in the aorta

**Figure 7:**
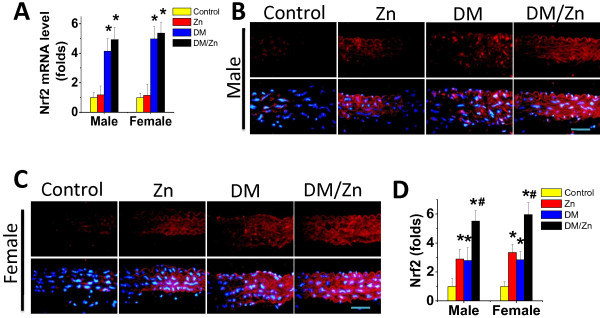
**Effects of Zn on aortic expression of Nrf2.** Aortic expression of Nrf2 was examined by real-time PCR for its mRNA level (**A**) and immunohistochemical staining for its protein expression (red) in aortic tunica media (**B** for male and **C** for female) with semi-quantitative analysis (**D** for both male and female).Data were presented as means ± SD (n = 6).*, p < 0.05 vs. Corresponding Control; #, p < 0.05 vs. Corresponding DM. Bar = 50 μM.

Aortic Nrf2 expression was examined by real-time qPCR for its mRNA level (Figure 7A) and immunofluorescent staining for its protein level (Figure 7B,C), followed by semi-quantitative analysis (Figure 7D). The qPCR analysis showed that diabetes significantly increased aortic expression of Nrf2 at mRNA levels; an effect was not seen in Zn-treated normal mice. Interestingly Zn treatment also did not significantly affect diabetes-induced Nrf2 mRNA expression. However, immunofluorescent staining showed that both Zn supplement and diabetes could significantly increase Nrf2 expression at the protein level (Figure 7B-D).As a result, the DM/Zn group, which had both the diabetes and the Zn treatment, had the highest aortic content of Nrf2 protein among all the groups. Furthermore, Figures 7B,C also show an increased accumulation of Nrf2 predominantly in the nuclei, suggesting the potential increase in its transcriptional function.

**Figure 8:**
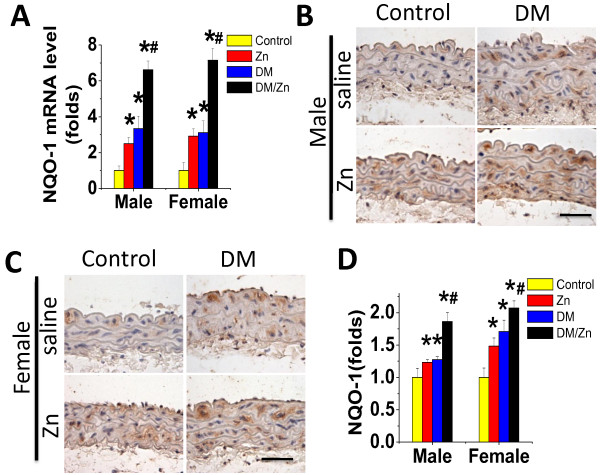
**Effects of Zn on aortic expression of Nrf2 downstream genes.** Aortic expression of Nrf2 down-stream genes NQO-1 expression was examined by real-time PCR at mRNA level (**A**) and immunohistochemical staining for protein expression in aortic tunica media (**B** for male and **C** for female) with semi-quantitative analysis (**D** for both male and female).Data were presented as means ± SD (n = 6).*, p < 0.05 vs. Corresponding Control; #, p < 0.05 vs. Corresponding DM. Bar = 50 μM.

To support the up-regulation of Nrf2 transcriptional function, qPCR analysis shows that one of Nrf2 downstream genes, NQO1, was significantly increased at its mRNA level in the aorta of Zn, DM, and DM/Zn groups with the highest expression in the DM/Zn group (Figure 8A). The up-regulated mRNA expression of NQO-1 was accompanied with a significant increase in its protein expression (Figure 8B-D). There was no gender difference for either NQO-1 mRNA expression or its protein expression.

### Up-regulation of MT mRNA and protein expression by Zn in the aorta

**Figure 9:**
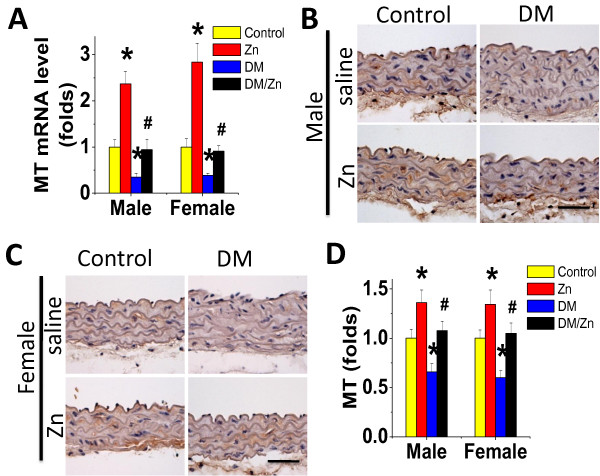
**Effects of Zn on aortic expression of MT.** Aortic expression of MT expression was examined by real-time PCR for its mRNA level (**A**) and immunohistochemical staining for its protein expression in aortic tunica media (**B** for male and **C** for female), followed with semi-quantitative analysis (**D** for both male and female).Data were presented as means ± SD (n = 6).*, p < 0.05 vs. Corresponding Control; #, p < 0.05 vs. Corresponding DM. Bar = 50 μM.

Since several studies have proposed the potential role of MT in Zn cardiac and renal protection against diabetes [18,23,24,29], we next examined the MT expression at both mRNA and protein levels in the aorta. Zn supplementation to control mice significantly increased the aortic expression of MT at mRNA level (Figure 9A), along with an increased expression of MT protein (Figure 9B-D) in the aorta of both male and female mice, compared with untreated control. However, diabetes significantly decreased the expression of MT mRNA and protein (Figure 9) while Zn treated diabetic mice preserved MT mRNA and protein expression levels comparable to control level (Figure 9).

## Discussions

In the present study, we have explored, for the first time, the protective effect of Zn supplementation on diabetes-induced pathogenic changes in the vessel, particularly in the aorta of genetic type 1 diabetic OVE26 mice. We found significant increases in aortic oxidative damage, inflammation, fibrosis and thickness in OVE26 mice, which was completely prevented by Zn treatment for 3 months. Mechanistic studies showed that the aortic protection of Zn treatment against diabetes-induced aortic pathogenesis was associated both with the up-regulation of Nrf2 protein expression and transcription, shown by the increased expression of Nrf2 down-stream gene NQO-1 at both mRNA and protein levels, and with the up-regulation of MT expression.

Zn plays important roles in the protection of vascular system from oxidative stress and damage. For instance, Zn deficiency in diabetic patients was found to associate with increased cardiovascular events [30], which may be related to the increased inflammatory response in system and vascular system [31-34]. Chronic inflammation plays a critical role for the development of various chronic pathogeneses [35-38]. The effects of chronic inflammation include induction of oxidative stress, apoptotic cell death, and abnormal cell proliferation, all which could contribute to the tissue structural and functional abnormalities [35-38]. In the present we demonstrated the induction of aortic inflammation, shown by increased expression of TNF-α, VACM-1 and PAI-1 in the aorta of DM, which was accompanied with increased aortic oxidative stress, apoptotic cell death, cell proliferation, and remodeling in DM group. All these pathogenic changes were prevented by Zn supplementation.

In contrast to Zn deficiency, Zn supplementation was found beneficial for the patients with various cardiovascular diseases [11,14,39-43]. Recently we also reported the aortic protection from diabetes-induced damage with sulforaphane in a type diabetic mouse model, in which, however, we have used streptozotocin (STZ) to induce diabetic mouse model [44], as used most frequently by others [45-48]. However, in STZ-induced diabetic animals STZ may have direct toxic effects on multiple organs [49]. Compared to STZ-induced diabetic mice, OVE26 mice exhibit more characteristics of human diabetic nephropathy, showing the time-dependent proteinuria [28,50,51]. Therefore, here we used OVE26 and age-matched WT mice to demonstrate the aortic protection by Zn supplementation from diabetes-induced pathogenic damages, including oxidative damage, inflammation and fibrotic response.

In the present study we used both male and female mice to compare whether there is gender difference for the pathogenic changes in the aorta. No difference was found either for the pathogenic change in the aorta induced by diabetes or the preventive effect of Zn on diabetes-induced pathogenic changes in the aorta. Interestingly a previous study compared the impact of gender on cardiac contractile response in ventricular myocytes from wild-type FVB and OVE26 mice at young (2 month) and older (11 month) age [52]. They found that OVE26 myocytes displayed reduced peak shortening (PS) and maximal velocity of shortening/relengthening (+/− dL/dt), and prolonged time-to-PS and time-to-90% relengthening, associated with higher resting intracellular Ca2+ levels and attenuated Ca(2+)-induced intracellular Ca2+ release compared with the FVB myocytes. Peak shortening and +/− dL/dt were smaller in female FVB groups when compared to the age-matched male counterparts. However, these gender differences were significant at 2 month old mice, but not at 11 month old mice. Therefore, mechanical differences existed between genders but were “cancelled off” by diabetic state. Nevertheless, a “female advantage” in ventricular function may still persist in young female diabetic subjects [52]. Therefore, the age of OVE26 mice used here may explain the lack of gender impact on diabetes-induced pathogenic changes and its protection by Zn since these mouse ages are 6 months old.

One important mechanism by which Zn protects the aorta from diabetes may be related to the up-regulation of Nrf2 in the aorta. Recently the important protection by Nrf2 from diabetes in various organs, including the heart and kidney, has been extensively reported [17,53,54]. There were several reports to indicate the possible induction of Nrf2 and its down-stream antioxidant genes by Zn [14,15,55]. However, here we provided the first evidence to indicate the Zn protection against diabetes-induced pathogenic changes in the aorta of diabetic mice treated with Zn for 3 months, probably associated with the up-regulation of Nrf2 expression and function.

One of the novel findings in the present study is the difference for Nrf2 expression in the aorta of mice in response to diabetes and Zn. Nrf2 protein level (about 3 fold increase) is significantly lower than mRNA level (about 6 fold increase) in diabetes group, suggesting the increase of Nrf2 degradation, resulting in only about 3 fold increase in protein level. In contrast, Zn treatment did not increase Nrf2 mRNA level, but increased Nrf2 protein level to about 3 fold higher than control, suggesting Zn’s stabilization of Nrf2, which also explain why in DM/Zn group the Nrf2 expression is about 6 fold, since it is the combined outcome of the diabetic induction of Nrf2 mRNA and Zn stabilization of Nrf2 protein.

How Zn stabilizes Nrf2 protein level remains unclear, however, there was an interesting report that supports this stabilization theory [55]. Chronic alcohol ingestion in rats decreases Zn levels and macrophage function in the alveolar space, which was accompanied with a decrease in Nrf2 nuclear binding capacity, i.e.: Nrf2 function decrease. When these rats were supplemented with Zn, Zn deficiency was corrected, resulting in a restoration of Nrf2 nuclear banding capacity along with a prevention of alcohol-induced macrophage function in the alveolar space of these rats [55]. This study suggests the requirement of Zn for maintaining normal function of Nrf2 in certain conditions.

However, a question is why the increased pathologic change was still observed in the aorta of OVE26 diabetic mice that also showed a significant increase in aortic Nrf2 expression. We assumed that the expression of Nrf2 and its downstream antioxidants in the aortas of diabetic mice is an adaptive response to diabetes. Therefore, the increased level in DM group that is significantly less than that in DM/Zn group may be not enough to rescue all injuries. However, although this adaptive response is unable to completely prevent, it should still protect certain levels of pathogenic damage induced by diabetes; otherwise these pathogenic changes would be more severe and appear earlier.

In addition, Zn protection from diabetes-induced aortic injury may also include other possible mechanisms. One most likely alternative mechanism may be the induction of MT since MT’s anti-oxidative protection from various conditions, including diabetes, has been extensively reported, even in our own laboratory [18,29,56-59]. Here we provide the first evidence that Zn induced, and diabetes decreased, the MT mRNA expression in the aorta, but Zn treatment can preserve the MT mRNA expression to control level in the OVE26 mice. In contrast to aortic Nrf2 or NQO-1 expression that was also up-regulated in diabetic group, aortic MT expression was significantly decreased in DM group, but not in DM/Zn group.

Although there was no much information regarding the role of MT in vascular function under diabetic condition, a few studies related it under other conditions also support the important role of MT in maintenance of normal vascular structure and function [60,61]. For instance, Zn protection from high-level glucose (HG), a diabetes feature on culture cardiomyocytes was observed recently [60]. The authors demonstrated that extracellular Zn2+ reduced cardiomyocyte contractile function in both HG and control groups, but enhanced relaxation function significantly in the HG group compared to controls. Most notably, a reduction in diastolic sarcomere length with increasing pacing frequencies, i.e., incomplete relaxation, was more pronounced in the HG compared to controls, but was normalized with extracellular Zn2+ application, implicating that the detrimental effect of HG on cardiomyocyte Ca2+ regulation can be amelioration by Zn^2+^[60]. Since MT was not measure in these cells, we do not know whether MT is involved in the beneficial effect of Zn. In another in vivo study, the effect of MT on collaterogenesis was demonstrated between mice with MT gene deletion and wild-type mice [61]. They found that mice defect in MT gene expression have impaired collateral flow recovery after the induction of acute hind limb ischemia. They further demonstrated that endothelial cells, smooth muscle cells, and macrophages that are known to be involved in collateral remodeling were dysfunctional in MT gene deficient mice. All these studies imply that MT plays an important role in maintaining the normal structure and function of vascular cells and vessels. Zn beneficial effect on vascular cells and/or vessels maybe related to MT expression, which need to further study in the future.

A potential limitation of the present study is that the most results are based on the immunohistochemical staining and real-time PCR assays. For the protein expression, this study will be greatly strengthened if we could have enough tissues to perform Western blotting assay. This will be warranted in the further study.

## Conclusions

In summary, we demonstrated here that Zn supplementation provides a significant protection against diabetes-induced pathogenic changes in the aorta without gender difference in the type 1 diabetic mouse model. The aortic protection by Zn against diabetes-induced pathogenic changes is associated with the up-regulation of both Nrf2 and MT expression.

## Abbreviations

3-NT: 3-nitrotyrosine; 4-HNE: 4-hydroxy-2-nonenal; CTGF: Connective tissue growth factor; DM: Diabetes; MT: Metallothionein; Nrf2: Nuclear factor (erythroid-derived 2)-like 2; NQO1: NADPH quinine oxidoreductase; PAI-1: Plasminogen activator inhibitor-1; TGFβ1: Transforming growth factor -β1; TNF-α: Tumor necrosis factor-alpha; VCAM-1: Vascular cell adhesion molecule 1; Zn: Zinc

## Competing interests

We declare that we have no conflict of interest.

## Authors’ contributions

XM, YW performed the majority of the laboratory work, which was initiated and designed by JS, YW, YZ, GS, QL and LC. LC, GS, QL and YW were the critical supervision of the experimental performance and critically involved in drafting, writing and revising the paper. All authors have read and approved the final manuscript.

## References

[B1] Chen H Goldberg MS Burnett RT Jerrett M Wheeler AJ Villeneuve PJ Long-term exposure to traffic-related air pollution and cardiovascular mortality Epidemiol 2013 24 1 35 43 10.1097/EDE.0b013e318276c005 23222554

[B2] Brunekreef B Beelen R Hoek G Schouten L Bausch-Goldbohm S Fischer P Armstrong B Hughes E Jerrett M Van Den Brandt P Effects of long-term exposure to traffic-related air pollution on respiratory and cardiovascular mortality in the Netherlands: the NLCS-AIR study Res Rep Health Eff Inst 2009 139 5 71 discussion 73–89 19554969

[B3] James J Soyibo AK Hurlock L Gordon-Strachan G Barton EN Cardiovascular risk factors in an eastern Caribbean island: prevalence of non-communicable chronic diseases and associated lifestyle risk factors for cardiovascular morbidity and mortality in the British Virgin Islands West Indian Med J 2012 61 4 429 436 10.7727/wimj.2012.173 23240481

[B4] Bell EJ Lutsey PL Windham BG Folsom AR Physical Activity and Cardiovascular Disease in African Americans in ARIC Med Sci Sports Exerc 2012 PMID: 23247714 10.1249/MSS.0b013e31827d87ec PMC3622814 23247714

[B5] Wannamethee SG Papacosta O Lawlor DA Whincup PH Lowe GD Ebrahim S Sattar N Do women exhibit greater differences in established and novel risk factors between diabetes and non-diabetes than men? The British Regional Heart Study and British Women’s Heart Health Study Diabetologia 2012 55 1 80 87 10.1007/s00125-011-2284-4 21861177

[B6] Wong NL Achike FI Gender discrimination in the influence of hyperglycemia and hyperosmolarity on rat aortic tissue responses to insulin Regul Pept 2010 163 1–3 113 119 20434492 10.1016/j.regpep.2010.04.003

[B7] Donahue RP Rejman K Rafalson LB Dmochowski J Stranges S Trevisan M Sex differences in endothelial function markers before conversion to pre-diabetes: does the clock start ticking earlier among women? The Western New York Study Diabetes Care 2007 30 2 354 359 10.2337/dc06-1772 17259507

[B8] Fukada T Yamasaki S Nishida K Murakami M Hirano T Zinc homeostasis and signaling in health and diseases: Zinc signaling J Biol Inorg Chem 2011 16 7 1123 1134 10.1007/s00775-011-0797-4 21660546 PMC3176402

[B9] McClain C Morris P Hennig B Zinc and endothelial function Nutrition 1995 11 1 Suppl 117 120 7749257

[B10] Hennig B Meerarani P Toborek M McClain CJ Antioxidant-like properties of zinc in activated endothelial cells J Am Coll Nutr 1999 18 2 152 158 10204831 10.1080/07315724.1999.10718843

[B11] Meerarani P Ramadass P Toborek M Bauer HC Bauer H Hennig B Zinc protects against apoptosis of endothelial cells induced by linoleic acid and tumor necrosis factor alpha Am J Clin Nutr 2000 71 1 81 87 10617950 10.1093/ajcn/71.1.81

[B12] Jenner A Ren M Rajendran R Ning P Huat BT Watt F Halliwell B Zinc supplementation inhibits lipid peroxidation and the development of atherosclerosis in rabbits fed a high cholesterol diet Free Radic Biol Med 2007 42 4 559 566 10.1016/j.freeradbiomed.2006.11.024 17275688

[B13] Alissa EM Bahijri SM Lamb DJ Ferns GA The effects of coadministration of dietary copper and zinc supplements on atherosclerosis, antioxidant enzymes and indices of lipid peroxidation in the cholesterol-fed rabbit Int J Exp Pathol 2004 85 5 265 275 10.1111/j.0959-9673.2004.00392.x 15379959 PMC2517529

[B14] Cortese MM Suschek CV Wetzel W Kroncke KD Kolb-Bachofen V Zinc protects endothelial cells from hydrogen peroxide via Nrf2-dependent stimulation of glutathione biosynthesis Free Radic Biol Med 2008 44 12 2002 2012 10.1016/j.freeradbiomed.2008.02.013 18355458

[B15] Ha KN Chen Y Cai J Sternberg P Jr Increased glutathione synthesis through an ARE-Nrf2-dependent pathway by zinc in the RPE: implication for protection against oxidative stress Invest Ophthalmol Vis Sci 2006 47 6 2709 2715 10.1167/iovs.05-1322 16723490

[B16] Lee JM Johnson JA An important role of Nrf2-ARE pathway in the cellular defense mechanism J Biochem Mol Biol 2004 37 2 139 143 10.5483/BMBRep.2004.37.2.139 15469687

[B17] Li B Liu S Miao L Cai L Prevention of diabetic complications by activation of Nrf2: diabetic cardiomyopathy and nephropathy Exp Diabetes Res 2012 2012 216512 22645602 10.1155/2012/216512 PMC3356887

[B18] Wang J Song Y Elsherif L Song Z Zhou G Prabhu SD Saari JT Cai L Cardiac metallothionein induction plays the major role in the prevention of diabetic cardiomyopathy by zinc supplementation Circulation 2006 113 4 544 554 10.1161/CIRCULATIONAHA.105.537894 16432057

[B19] Islam MS Du Loots T Diabetes, metallothionein, and zinc interactions: a review Biofactors 2007 29 4 203 212 10.1002/biof.5520290404 18057551

[B20] Yang J Cherian MG Protective effects of metallothionein on streptozotocin-induced diabetes in rats Life Sci 1994 55 1 43 51 10.1016/0024-3205(94)90080-9 8015348

[B21] Cai L Wang Y Zhou G Chen T Song Y Li X Kang YJ Attenuation by metallothionein of early cardiac cell death via suppression of mitochondrial oxidative stress results in a prevention of diabetic cardiomyopathy J Am Coll Cardiol 2006 48 8 1688 1697 17045908 10.1016/j.jacc.2006.07.022

[B22] Dong F Li Q Sreejayan N Nunn JM Ren J Metallothionein prevents high-fat diet induced cardiac contractile dysfunction: role of peroxisome proliferator activated receptor gamma coactivator 1alpha and mitochondrial biogenesis Diabetes 2007 56 9 2201 2212 10.2337/db06-1596 17575086

[B23] Ohly P Dohle C Abel J Seissler J Gleichmann H Zinc sulphate induces metallothionein in pancreatic islets of mice and protects against diabetes induced by multiple low doses of streptozotocin Diabetologia 2000 43 8 1020 1030 10.1007/s001250050009 10990080

[B24] Tang Y Yang Q Lu J Zhang X Suen D Tan Y Jin L Xiao J Xie R Rane M Zinc supplementation partially prevents renal pathological changes in diabetic rats J Nutr Biochem 2010 21 3 237 246 10.1016/j.jnutbio.2008.12.010 19369054

[B25] Yang J Tan Y Zhao F Ma Z Wang Y Zheng S Epstein PN Yu J Yin X Zheng Y Angiotensin II plays a critical role in diabetic pulmonary fibrosis most likely via activation of NADPH oxidase-mediated nitrosative damage Am J Physiol Endocrinol Metab 2011 301 1 E132 E144 10.1152/ajpendo.00629.2010 21487074

[B26] Cui W Li B Bai Y Miao X Chen Q Sun W Tan Y Luo P Zhang C Zheng S Potential role for Nrf2 activation in the therapeutic effect of MG132 on diabetic nephropathy in OVE26 diabetic mice Am J Physiol Endocrinol Metab 2013 304 1 E87 E99 10.1152/ajpendo.00430.2012 23132297

[B27] Epstein PN Overbeek PA Means AR Calmodulin-induced early-onset diabetes in transgenic mice Cell 1989 58 6 1067 1073 10.1016/0092-8674(89)90505-9 2673540

[B28] Zheng S Noonan WT Metreveli NS Coventry S Kralik PM Carlson EC Epstein PN Development of late-stage diabetic nephropathy in OVE26 diabetic mice Diabetes 2004 53 12 3248 3257 10.2337/diabetes.53.12.3248 15561957

[B29] Ozcelik D Naziroglu M Tuncdemir M Celik O Ozturk M Flores-Arce MF Zinc supplementation attenuates metallothionein and oxidative stress changes in kidney of streptozotocin-induced diabetic rats Biol Trace Elem Res 2012 150 1–3 342 349 23054862 10.1007/s12011-012-9508-4

[B30] Soinio M Marniemi J Laakso M Pyorala K Lehto S Ronnemaa T Serum zinc level and coronary heart disease events in patients with type 2 diabetes Diabetes Care 2007 30 3 523 528 10.2337/dc06-1682 17327315

[B31] Reiterer G MacDonald R Browning JD Morrow J Matveev SV Daugherty A Smart E Toborek M Hennig B Zinc deficiency increases plasma lipids and atherosclerotic markers in LDL-receptor-deficient mice J Nutr 2005 135 9 2114 2118 16140885 10.1093/jn/135.9.2114

[B32] Shen H Oesterling E Stromberg A Toborek M MacDonald R Hennig B Zinc deficiency induces vascular pro-inflammatory parameters associated with NF-kappaB and PPAR signaling J Am Coll Nutr 2008 27 5 577 587 18845708 10.1080/07315724.2008.10719741

[B33] Tomat AL Costa Mde L Arranz CT Zinc restriction during different periods of life: influence in renal and cardiovascular diseases Nutrition 2011 27 4 392 398 10.1016/j.nut.2010.09.010 21074972

[B34] De Oliveira Otto MC Alonso A Lee DH Delclos GL Bertoni AG Jiang R Lima JA Symanski E Jacobs DR Jr Nettleton JA Dietary intakes of zinc and heme iron from red meat, but not from other sources, are associated with greater risk of metabolic syndrome and cardiovascular disease J Nutr 2012 142 3 526 533 10.3945/jn.111.149781 22259193 PMC3278268

[B35] Simon DI Inflammation and vascular injury: basic discovery to drug development Circ J 2012 76 8 1811 1818 10.1253/circj.CJ-12-0801 22785436 PMC4090145

[B36] Ishigami N Isoda K Adachi T Niida T Kujiraoka T Hakuno D Kondo H Kusuhara M Ohsuzu F Deficiency of CuZn superoxide dismutase promotes inflammation and alters medial structure following vascular injury J Atheroscler Thromb 2011 18 11 1009 1017 10.5551/jat.9324 21946535

[B37] Puntmann VO Taylor PC Mayr M Coupling vascular and myocardial inflammatory injury into a common phenotype of cardiovascular dysfunction: systemic inflammation and aging - a mini-review Gerontology 2011 57 4 295 303 10.1159/000316577 20551624

[B38] Davis C Fischer J Ley K Sarembock IJ The role of inflammation in vascular injury and repair J Thromb Haemost 2003 1 8 1699 1709 10.1046/j.1538-7836.2003.00292.x 12911580

[B39] Zhang D Li Y Zhu T Zhang F Yang Z Miao D Zinc supplementation results in improved therapeutic potential of bone marrow-derived mesenchymal stromal cells in a mouse ischemic limb model Cytotherapy 2011 13 2 156 164 10.3109/14653249.2010.512633 20839997

[B40] Heidarian E Amini M Parham M Aminorroaya A Effect of zinc supplementation on serum homocysteine in type 2 diabetic patients with microalbuminuria Rev Diabet Stud 2009 6 1 64 70 10.1900/RDS.2009.6.64 19557297 PMC2712914

[B41] Hashemipour M Kelishadi R Shapouri J Sarrafzadegan N Amini M Tavakoli N Movahedian-Attar A Mirmoghtadaee P Poursafa P Effect of zinc supplementation on insulin resistance and components of the metabolic syndrome in prepubertal obese children Hormones (Athens) 2009 8 4 279 285 20045801 10.14310/horm.2002.1244

[B42] Parham M Amini M Aminorroaya A Heidarian E Effect of zinc supplementation on microalbuminuria in patients with type 2 diabetes: a double blind, randomized, placebo-controlled, cross-over trial Rev Diabet Stud 2008 5 2 102 109 10.1900/RDS.2008.5.102 18795212 PMC2556442

[B43] Bao B Prasad AS Beck FW Snell D Suneja A Sarkar FH Doshi N Fitzgerald JT Swerdlow P Zinc supplementation decreases oxidative stress, incidence of infection, and generation of inflammatory cytokines in sickle cell disease patients Transl Res 2008 152 2 67 80 10.1016/j.trsl.2008.06.001 18674741

[B44] Miao X Bai Y Su W Cui W Xin Y Wang Y Tan Y Miao L Fu Y Su G Sulforaphane prevention of diabetes-induced aortic damage was associated with the up-regulation of Nrf2 and its down-stream antioxidants Nutr Metab (Lond) 2012 9 1 84 10.1186/1743-7075-9-84 22978402 PMC3495894

[B45] Prevost G Bulckaen H Gaxatte C Boulanger E Beraud G Creusy C Puisieux F Fontaine P Structural modifications in the arterial wall during physiological aging and as a result of diabetes mellitus in a mouse model: are the changes comparable? Diabetes Metab 2011 37 2 106 111 10.1016/j.diabet.2010.08.005 21144786

[B46] Nuno DW Lamping KG The role of rho kinase in sex-dependent vascular dysfunction in type 1 diabetes Exp Diabetes Res 2010 2010 176361 20368772 10.1155/2010/176361 PMC2846338

[B47] Sasaki N Yamashita T Takaya T Shinohara M Shiraki R Takeda M Emoto N Fukatsu A Hayashi T Ikemoto K Augmentation of vascular remodeling by uncoupled endothelial nitric oxide synthase in a mouse model of diabetes mellitus Arterioscler Thromb Vasc Biol 2008 28 6 1068 1076 10.1161/ATVBAHA.107.160754 18403727

[B48] Uemura S Matsushita H Li W Glassford AJ Asagami T Lee KH Harrison DG Tsao PS Diabetes mellitus enhances vascular matrix metalloproteinase activity: role of oxidative stress Circ Res 2001 88 12 1291 1298 10.1161/hh1201.092042 11420306

[B49] Wold LE Ren J Streptozotocin directly impairs cardiac contractile function in isolated ventricular myocytes via a p38 map kinase-dependent oxidative stress mechanism Biochem Biophys Res Commun 2004 318 4 1066 1071 10.1016/j.bbrc.2004.04.138 15147982

[B50] Teiken JM Audettey JL Laturnus DI Zheng S Epstein PN Carlson EC Podocyte loss in aging OVE26 diabetic mice Anat Rec (Hoboken) 2008 291 1 114 121 10.1002/ar.20625 18085629

[B51] Zheng S Carlson EC Yang L Kralik PM Huang Y Epstein PN Podocyte-specific overexpression of the antioxidant metallothionein reduces diabetic nephropathy J Am Soc Nephrol 2008 19 11 2077 2085 10.1681/ASN.2007080967 18632844 PMC2573005

[B52] Zhang X Ye G Duan J Chen AF Ren J Influence of gender on intrinsic contractile properties of isolated ventricular myocytes from calmodulin-induced diabetic transgenic mice Endocr Res 2003 29 2 227 236 10.1081/ERC-120022318 12856810

[B53] He X Kan H Cai L Ma Q Nrf2 is critical in defense against high glucose-induced oxidative damage in cardiomyocytes J Mol Cell Cardiol 2009 46 1 47 58 10.1016/j.yjmcc.2008.10.007 19007787

[B54] De Haan JB Nrf2 activators as attractive therapeutics for diabetic nephropathy Diabetes 2011 60 11 2683 2684 10.2337/db11-1072 22025774 PMC3198074

[B55] Mehta AJ Joshi PC Fan X Brown LA Ritzenthaler JD Roman J Guidot DM Zinc supplementation restores PU.1 and Nrf2 nuclear binding in alveolar macrophages and improves redox balance and bacterial clearance in the lungs of alcohol-fed rats Alcohol Clin Exp Res 2011 35 8 1519 1528 21447000 10.1111/j.1530-0277.2011.01488.x PMC3128659

[B56] Liang Q Carlson EC Donthi RV Kralik PM Shen X Epstein PN Overexpression of metallothionein reduces diabetic cardiomyopathy Diabetes 2002 51 1 174 181 11756338 10.2337/diabetes.51.1.174

[B57] Song Y Wang J Li Y Du Y Arteel GE Saari JT Kang YJ Cai L Cardiac metallothionein synthesis in streptozotocin-induced diabetic mice, and its protection against diabetes-induced cardiac injury Am J Pathol 2005 167 1 17 26 10.1016/S0002-9440(10)62949-5 15972948 PMC1603431

[B58] Cai L Suppression of nitrative damage by metallothionein in diabetic heart contributes to the prevention of cardiomyopathy Free Radic Biol Med 2006 41 6 851 861 10.1016/j.freeradbiomed.2006.06.007 16934665

[B59] Zhou G Li X Hein DW Xiang X Marshall JP Prabhu SD Cai L Metallothionein suppresses angiotensin II-induced nicotinamide adenine dinucleotide phosphate oxidase activation, nitrosative stress, apoptosis, and pathological remodeling in the diabetic heart J Am Coll Cardiol 2008 52 8 655 666 10.1016/j.jacc.2008.05.019 18702970

[B60] Yi T Cheema Y Tremble SM Bell SP Chen Z Subramanian M LeWinter MM VanBuren P Palmer BM Zinc-induced cardiomyocyte relaxation in a rat model of hyperglycemia is independent of myosin isoform Cardiovasc Diabetol 2012 11 135 10.1186/1475-2840-11-135 23116444 PMC3537566

[B61] Zbinden S Wang J Adenika R Schmidt M Tilan JU Najafi AH Peng X Lassance-Soares RM Iantorno M Morsli H Metallothionein enhances angiogenesis and arteriogenesis by modulating smooth muscle cell and macrophage function Arterioscler Thromb Vasc Biol 2010 30 3 477 482 10.1161/ATVBAHA.109.200949 20056912

